# Asymptomatic stage I sarcoidosis complicated by pulmonary tuberculosis: a case report

**DOI:** 10.1186/1752-1947-2-226

**Published:** 2008-07-07

**Authors:** Georgios S Papaetis, Angelos Pefanis, Solon Solomon, Ioannis Tsangarakis, Dora Orphanidou, Apostolos Achimastos

**Affiliations:** 1 3rd Department of Medicine, University of Athens, Medical School, 'Sotiria' General Hospital, Athens, Greece

## Abstract

**Introduction:**

Sarcoidosis is a multisystem granulomatous disorder characterized pathologically by the presence of non-caseating granulomas in involved tissues. Depressed cellular immunity predisposes patients to infections with certain intracellular organisms, mostly fungi, *Mycobacterium tuberculosis *and *Nocardia *species. As these infections are mainly insidious and difficult to differentiate from the underlying disease, a possible misdiagnosis may lead to fatal complications for the patient.

**Case presentation:**

We present a case of a 67-year-old woman with undiagnosed asymptomatic stage I sarcoidosis for at least 8 years before her admission and a 1-month history of fever, exertional dyspnea and dry cough, in whom pulmonary tuberculosis was documented.

**Conclusion:**

This case highlights the need for great vigilance among physicians in order to rule out any possible infection before establishing the diagnosis of sarcoidosis.

## Introduction

Sarcoidosis is a multisystem granulomatous disorder of uncertain etiology, characterized pathologically by the presence of non-caseating granulomas in involved tissues [1]. Approximately half of cases are diagnosed incidentally by radiographic abnormalities on a routine chest radiograph. Depressed cellular immunity predisposes patients to opportunistic infections with certain intracellular organisms, mostly fungi, *Mycobacterium tuberculosis *and *Nocardia *species. Moreover, the prevalence of these infecting organisms in patients with early stage untreated disease is rather infrequent. As these infections are mainly insidious and difficult to differentiate from the underlying disease, a possible misdiagnosis may lead to fatal complications for the patient [2].

We describe a patient with undiagnosed asymptomatic stage I sarcoidosis for at least 8 years before her admission and a 1-month history of fever, exertional dyspnea and dry cough, in whom pulmonary tuberculosis (TB) was documented. This case highlights the high index of suspicion required in order to identify any possible infection before the diagnosis of sarcoidosis is established.

## Case presentation

A 67-year-old woman presented to the hospital complaining of fever, shortness of breath and dry cough during the previous month. She had been treated for presumed bronchitis with wide-spectrum antibiotics without response and her complaints had gradually worsened. Her past medical history was significant for bilateral hilar lymphadenopathy, which was incidentally diagnosed on a routine chest radiograph 8 years previously, a finding that was confirmed, together with right paratracheal node enlargement, by a chest computed tomography (CT) scan. She had then undergone a non-diagnostic bronchoscopy and was advised to repeat the chest CT scan after 6 months, advice she ignored. She had never smoked and she had not taken any medication in the past. She had no environmental or occupational history of beryllium or other metal exposure. She had never traveled outside Greece. She had never had a tuberculin test.

**Figure 1:**
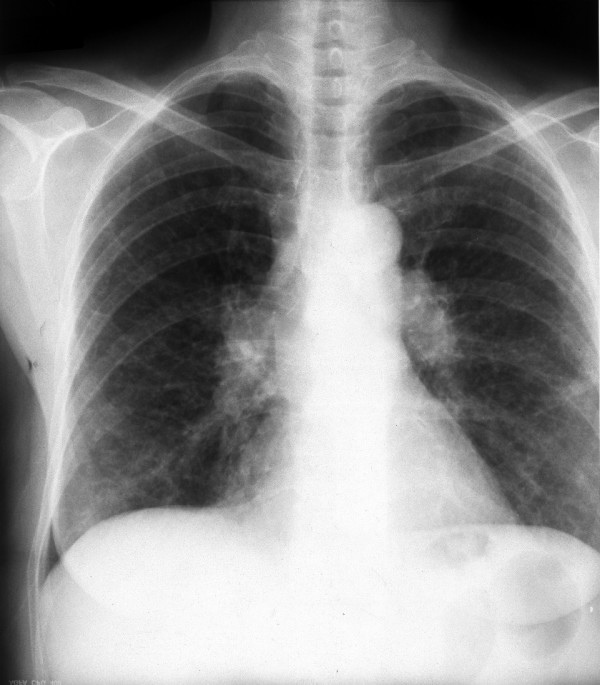
Chest X-ray showing bilateral hilar lymphadenopathy together with lower bilateral interstitial lung densities.

**Figure 2:**
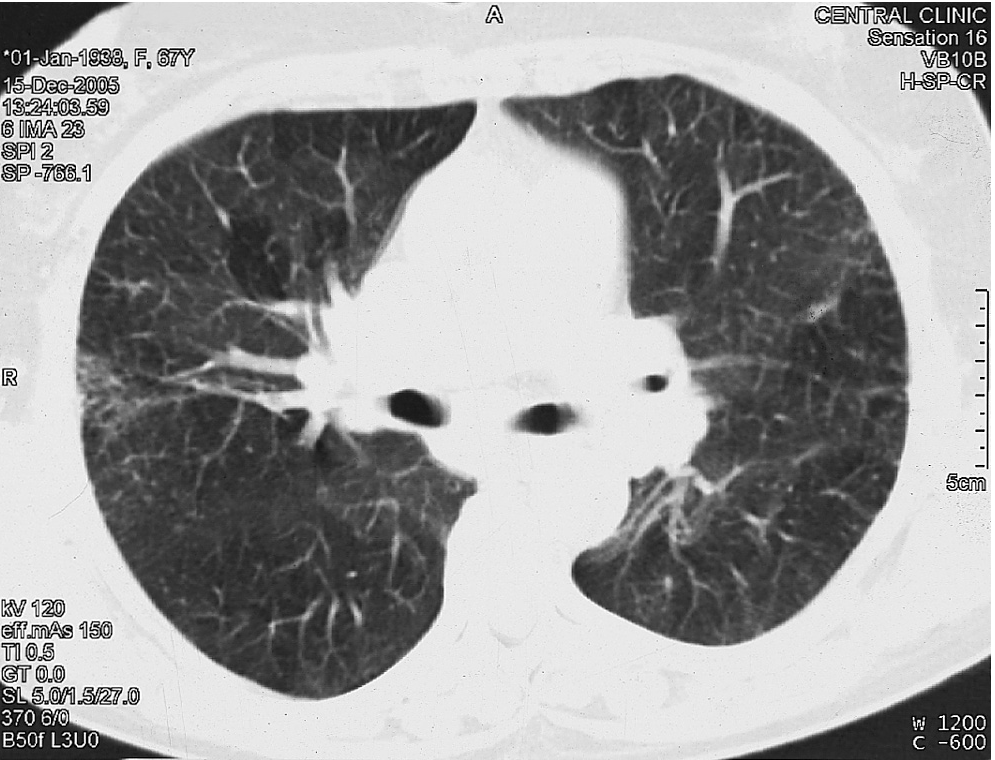
Computed tomography scan of the chest with bilateral mediastinal lymphadenopathy and bilateral interstitial lung opacities.

On physical examination, the patient appeared to be in good condition, mildly dyspneic with 22 breaths per minute, a temperature of 39.4°C, blood pressure of 110/70 mmHg and a heart rate of 100 beats per minute. Apart from mild bilateral inspiratory fine crackles in the lower lung fields, no other physical abnormalities were observed. There was no skin involvement. Laboratory investigations showed normocytic normochromic anemia (hemoglobin 11.8 g/dl), white blood count 6370/mm^3 ^(neutrophils 67%), erythrocyte sedimentation rate 95 mm and C-reactive protein 90 mg/l (normal value <3 mg/l). Serum electrolyte levels and renal function indices were normal. A mild decrease in albumin levels was observed on serum protein electrophoresis. Serum concentrations of angiotensin-converting enzyme (ACE) were normal, as was a 24-hour urinary calcium excretion analysis. Arterial gas testing (while the patient was breathing in room air) indicated PaO_2 _70 mmHg, PaCO_2 _31 mmHg, pH 7.47 and bicarbonate 22.2 mmol/l. The tuberculin skin test was positive (20 mm). A chest X-ray on admission disclosed bilateral hilar lymphadenopathy together with bilateral interstitial lung densities in the lower lung fields (Figure 1). A chest CT scan that was performed 3 days after her admission disclosed bilateral interstitial opacities in the middle and lower lung lobes and mediastinal lymphadenopathy, which was unaltered compared with that observed 8 years ago (Figure 2).

An ophthalmologic evaluation was normal. The patient underwent bronchoscopy, which revealed slight mucosal edema in the bronchial tree. All endobronchial biopsies disclosed non-specific inflammation. Transbronchial biopsies were not performed because of the patient's intolerance. The bronchoalveolar lavage (BAL) cell differential count was total cell count 37 × 10^4^/ml, neutrophils 2.8%, lymphocytes 61.2%, macrophages 34.5% and eosinophils 1.3%. The CD4 to CD8 ratio was 2.25 while the number of CD4 and CD8 cells was within normal levels. Multiple blood and urine cultures as well as bronchial washing cultures were negative for bacteria and fungi. Results from sputum, gastric fluid and BAL Ziehl-Neelsen staining were negative. Serology studies for human immunodeficiency virus (HIV), *Brucella *species, *Legionella *species, *Coxiella burnetti*, *Chlamydia *species, *Mycoplasma *species, *Leismania *species and *Toxoplasma *species were negative. A venereal disease research laboratory test was also negative. Antinuclear antibodies were 1/80 positive while anti-double-stranded DNA antibodies, anti-RO, anti-LA, antineutrophil cytoplasmic antibodies and antimitochondrial antibodies were all negative. The patient underwent mediastinoscopy, and histological examination from the biopsy of a right paratracheal lymph node showed a non-caseating granuloma formation compatible with sarcoidosis. Routine tissue bacterial, fungal and acid bacilli cultures were negative.

**Figure 3:**
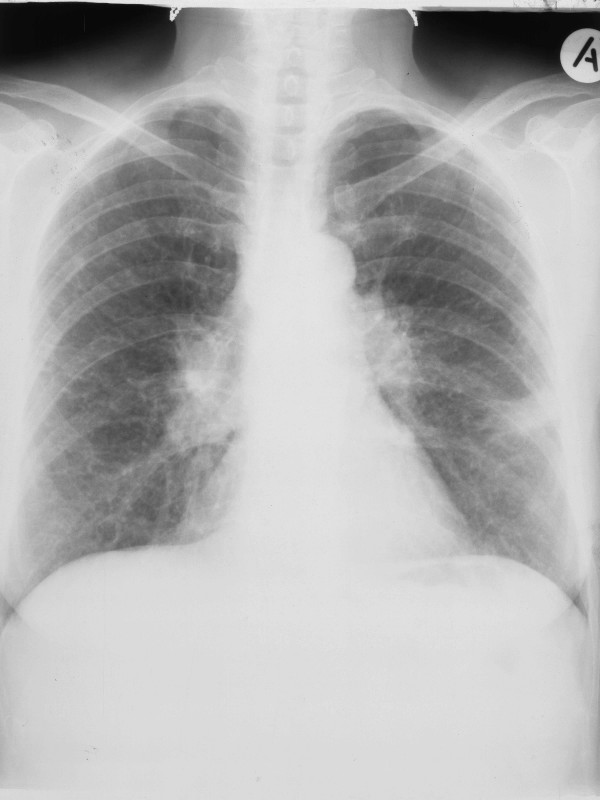
Chest X-ray showing extended bilateral interstitial lung densities and left lower lung field consolidation.

The patient received broad-spectrum antibiotic treatment without any response, while her dyspnea progressively increased and she was supported with oxygen therapy. A chest X-ray disclosed extended interstitial lung densities in both lungs as well as consolidation in the left lower lung field (Figure 3). As she was clinically and radiologically deteriorating, treatment with the standard anti-TB drug regimen (isoniazid 300 mg daily, rifampin 600 mg daily, pyrazinamide 2 g daily and ethambutol 1 g daily) was started in addition to prednisolone 1 mg/kg daily. The patient's symptoms moderately improved but her fever continued to spike. During the second week of anti-TB therapy, initial BAL cultures turned out to be positive for *M. tuberculosis *and glucocorticoids were stopped. During the fourth week of treatment the fever declined and the patient became apyrexial 1 week later. After 2 months of therapy, pyrazinamide and ethambutol were stopped and a 7-month continuation regimen with isoniazide and rifampin was administered. During a 9 month period of follow-up, the patient continued to have the same chronic mediastinal lymphadenopathy with no parenchymal involvement and normal pulmonary function tests.

## Discussion

The combination of chronic bilateral hilar lymphadenopathy, the histopathological detection of non-caseating granulomas and the exclusion of other diseases with similar presentation suggested the diagnosis of sarcoidosis in our patient, although a definite diagnostic test does not exist. Approximately 75% of patients with stage I disease may experience regression of hilar nodes in 1 to 3 years while 10% develop chronic enlargement that exists for 10 years or more [3]. Cutaneous involvement is seen in up to 20% of patients with sarcoidosis, with maculopapular eruption being the most common subacute lesion [4]. The association of sarcoidosis with TB still remains complex, although it has been thoroughly studied. TB has been described as both preceding and co-existing with sarcoidosis as well as being an opportunistic infection in patients with a documented disease who mostly follow corticosteroid therapy, as glucocorticoids greatly depress the disordered cell-mediated immunity observed [5,6].

The clinical and radiological deterioration of our patient despite treatment with wide-spectrum antibiotics, combined with the interstitial pattern in chest CT scans, the negative bacterial, fungal cultures and serology, as well as the negative Ziehl-Neelsen staining in all samples collected, suggested a possible disease progression. Ground-glass opacification, rather than alveolitis, has been shown to be associated with sarcoid granulomas in these patients [7]. On the other hand, both serum ACE levels and BAL CD4 and CD8 cell numbers were not indicative of sarcoidosis, and the tuberculin test created a dilemma as to whether we should start glucocorticoids in our patient, since the acid-bacilli cultures were pending [8]. Epidemiological studies examining the prevalence of positive tuberculin tests in Greece have been organized mainly in Hellenic Army recruits. According to the most recent data published, there has been a decline of tuberculin test positivity from 14.2% in 1981 to 6.8% in 1991 and to 3.9% in November 2005–February 2006 [9]. As our patient's condition had seriously deteriorated, the concurrent administration of glucocorticoids with anti-TB treatment may have had a beneficial effect [10].

Although interstitial parenchymal opacities are not common in HIV-negative patients with pulmonary TB, Chin et al. have recently reported that in a series of 22 patients with fever of unknown origin finally diagnosed with mycobacterial infection, including 19 TB patients, 41% of them had an interstitial pattern on chest radiographs [11].

## Conclusion

We have presented the case of a patient with asymptomatic stage I sarcoidosis who developed pulmonary TB, and have emphasized the diagnostic dilemmas that may occur when these conditions coexist. Although infections with certain intracellular organisms, including *M. tuberculosis*, are probably infrequent in patients with sarcoidosis, there needs to be a greater awareness among physicians so as to rule out any possible infection before establishing the diagnosis of sarcoidosis. The initiation of steroid therapy in patients with an underlying infection may accentuate any possible life-threatening complications.

## Abbreviations

ACE: Angiotensin-converting enzyme; BAL: Bronchoalveolar lavage; CT: Computed tomography; HIV: Human immunodeficiency virus; TB: Tuberculosis.

## Competing interests

The authors declare that they have no competing interests.

## Authors' contributions

Each author participated equally in the diagnosis, treatment and follow-up of the patient. All authors read and approved the final manuscript.

## Consent

Written informed consent was obtained from the patient for publication of this case report and accompanying images. A copy of the written consent is available for review by the Editor-in-Chief of the journal.
